# Body Mass Index, Waist Circumference, Body Adiposity Index, and Risk for Type 2 Diabetes in Two Populations in Brazil: General and Amerindian

**DOI:** 10.1371/journal.pone.0100223

**Published:** 2014-06-17

**Authors:** Rafael de Oliveira Alvim, Carlos Alberto Mourao-Junior, Camila Maciel de Oliveira, José E. Krieger, José G. Mill, Alexandre C. Pereira

**Affiliations:** 1 Laboratory of Genetics and Molecular Cardiology, Heart Institute (InCor), University of Sao Paulo Medical School, Sao Paulo, Brazil; 2 Department of Physiology, Federal University of Juiz de Fora, São Pedro, Juiz de Fora - MG, Brazil; 3 Department of Physiology, Federal University of Espírito Santo, Espírito Santo, Brazil; University of Catanzaro Magna Graecia, Italy

## Abstract

**Objective:**

The use of the anthropometric indices of adiposity, especially body mass index and waist circumference in the prediction of diabetes mellitus has been widely explored. Recently, a new body composition index, the body adiposity index was proposed. The aim of this study was to compare the effectiveness of body mass index, waist circumference, and body adiposity index in the risk assessment for type 2 diabetes mellitus.

**Design and methods:**

A total of 1,572 individuals from the general population of Vitoria City, Brazil and 620 Amerindians from the Aracruz Indian Reserve, Brazil were randomly selected. BMI, waist circumference, and BAI were determined according to a standard protocol. Type 2 diabetes mellitus was diagnosed by the presence of fasting glucose ≥126 mg/dL or by the use of antidiabetic drugs.

**Results:**

The area under the curve was similar for all anthropometric indices tested in the Amerindian population, but with very different sensitivities or specificities. In women from the general population, the area under the curve of waist circumference was significantly higher than that of the body adiposity index. Regarding risk assessment for type 2 diabetes mellitus, the body adiposity index was a better risk predictor than body mass index and waist circumference in the Amerindian population and was the index with highest odds ratio for type 2 diabetes mellitus in men from the general population, while in women from the general population waist circumference was the best risk predictor.

**Conclusion:**

Body adiposity index was the best risk predictor for type 2 diabetes mellitus in the Amerindian population and men from the general population. Our data suggest that the body adiposity index is a useful tool for the risk assessment of type 2 diabetes mellitus in admixture populations.

## Introduction

Diabetes Mellitus (DM) is a multifactorial metabolic disease associated with several conditions, including physical inactivity, genetic predisposition, poor nutrition, and obesity [1], [2]. The association between DM (especially T2DM) and obesity is well established: obese individuals have a twentyfold risk of developing diabetes compared with individuals of normal weight [3].

The strong association between obesity and cardiometabolic disorders motivated the development of several techniques used to determine body adiposity, such as body mass index (BMI), waist circumference (WC), and waist-hip ratio (WHR) [4], [5], BMI being a general obesity indicator and both WC and WHR abdominal obesity indicators [6]. Recently, Bergman et al [7] proposed the body adiposity index (BAI) as an alternative to BMI to possibly overcome deficiencies in the latter method in assessing overweight and obesity. However, unlike both BMI and WC [5], the role of BAI as a risk marker for DM is unclear. Thus, it is relevant to compare the effectiveness of BAI with both WC and BMI in the assessment of risk for DM in different populations.

Therefore, the aim of this study was to compare which of the three measurements (BMI, WC, or BAI) is a better risk predictor for T2DM in the general and Amerindian populations of Brazil.

## Methods

### Subjects

A study of risk factors for cardiovascular diseases was performed in the urban population of Vitoria, Brazil, using the WHO-MONICA project guidelines [8]. The study design was based on cross-sectional research methodology and was developed by surveying and analyzing socioeconomic and health data in a probabilistic sample of residents from the municipality of Vitoria, Espírito Santo, Brazil. The sampling plan had the objective of ensuring that the research would be socioeconomically, geographically, and demographically representative of the residents of this municipality. The resident population aged 25–64 years in the city of Vitoria was studied. According to the census carried out by the IBGE Foundation in 1996, the resident population of Vitoria included 265,874 inhabitants. The sampling was performed in four stages: by district, IBGE census sector, drawing lots to choose homes, and drawing lots to choose an individual from each home. The survey was conducted with just one resident from the home that was selected, within the age group of the study. The draw was carried out by using a randomization mechanism. We selected 2,268 residential homes located in Vitoria and visited them. We explained the purposes of the research to the individual selected at each of these homes and invited the individual to participate in the study, after obtaining his or her written consent. The selected individuals were asked to attend the Cardiovascular Investigation Clinic of the University Hospital for tests to be performed on the following day. Of the total sample, 1,572 individuals attended (715 males and 857 females).

Aiming to replicate the data found in the population of Vitoria (WHO-MONICA project guidelines), we also used data from a cross-sectional study of risk factors for cardiovascular diseases that was carried out in two Indian groups (Guarani and Tupinikin) living on the Aracruz Indian Reserve, Espírito Santo State, on the southeast Brazilian coast. All individuals (n = 620; 292 males and 328 females) aged 20 years or more were eligible for the study. During small meetings in each of the five small settlements within the Reserve, the eligible individuals were invited to participate in the study. Data were collected from February 2003 to April 2004, and 670 (80.3% of the eligible population) attended the local health unit to undergo clinical and laboratory examinations necessary to identify cardiovascular risk factors.

This study was approved by the ethics committee for Research on Human Subjects of the Espírito Santo Federal University and National Ethics Committee for Human Research (CONEP Register Number 4599).

### Anthropometrical Investigations

Anthropometric parameters were measured according to a standard protocol [9]. Body weight was measured on a calibrated scale, to the nearest 0.1 kg. Height was measured using a wall-mounted stadiometer, to the nearest 0.5 cm. WC was measured at the mean point between the lowest rib margin and the iliac crest with the subject standing and at the maximum point of normal expiration. Hip circumference was measured to the nearest 0.1 cm around the thighs, at the height of the greater trochanter, in the standing position. BMI was calculated as body weight (kg) divided by height squared (m^2^). BAI was calculated using hip circumference and height (BAI = [hip (cm)/height (m)^1.5^]−18) [7].

### Biochemical Measurement

Fasting glucose was evaluated using standard techniques applied to 12-h fasting blood samples [10]. We adopted an epidemiological classification of DM [11]. Thus, T2DM was diagnosed by the presence of fasting glucose ≥126 mg/dL or by the use of antidiabetic drugs, except insulin.

### Statistics Analyses

Categorical variables are presented as percentages, whereas continuous variables are presented as mean ± standard deviation. To evaluate the performance models, a receiver operating characteristic (ROC) curve was built and the AUC was used to measure the discriminatory power for T2DM. Areas under the ROC curves between the markers were compared using a parametric method, with GraphROC for Windows software [12]. The optimal cutoff points for BAI, BMI, and WC were established based on the highest combination of sensitivity and specificity. In addition, the positive and negative predictive values (PPV and NPV, respectively) for each anthropometric index were determined. Logistic regression analyses were used to assess the risk association between the different measurements (WC, BMI, and BAI) and T2DM. To standardize measures, all indices were transformed to *z* scores [13]. All analyses were adjusted by age, mean blood pressure, and total cholesterol. The adjustment of the models was verified using the *Hosmer-Lemeshow test* (p>0.05). Statistical analyses were carried out using SPSS (version 20) software (Chicago, IL, USA), with the level of significance set at 5%.

## Results

### Amerindian Population

Demographic data related to age, BMI, BAI, WC, fasting glucose, and T2DM percentage stratified by sex are summarized in Table 1.

**Table 1:** Characteristics of subjects in the Amerindian and General population.

	Amerindians		General Population		
Characteristics	Men	Women	Men		Women
**n**	292	328	715		857
**Age, years**	37.3±14.6	37.1±14.5	44.7±10.9		44.8±10.8
**BMI, Kg/m^2^**	24.6±3.6	26.2±5.0	25.9±4.0		26.6±5.5
**BAI**	21.7±4.924	29.9±6.4	26.0±3.5		32.9±5.6
**Waist Circunference, cm**	83.5±9.5	84.5±11.6	89.2±10.9		83.6±12.9
**Fasting Glucose, mg/dL**	92.4±17.0	89.7±18.1	105.6±28.5		104.4±34.6
**NIDDM, %**	2.4%	2.7%	6.9%		8.6%

BMI, body mass index; BAI, body adiposity index; T2DM, type 2 diabetes mellitus.

Cutoffs, sensitivity, specificity, PPV, NPV, and AUC are reported in Table 2. In this population, although the overall accuracy of tested measures in the risk assessment for T2DM were not significantly different between men and women, important differences regarding sensitivity, specificity, and predictive values were observed when comparing men and women for all three of the measures tested. In addition, as expected, differences in the optimum cutoff values between men and women were also observed. The best nominal predictor of T2DM, in both Amerindian men and women, was BAI, although with a greater accuracy in men (0.84×0.77).

**Table 2:** Determination of the optimal cutoffs values and AUC for BMI, BAI and WC in Amerindian and General population.

Population	Antropometric index	Cutoffs	Sensitivity	Specificity	PPV	NPV	AUC (95%CI)	*p* value
**Amerindian**								
	**Men**							
	BMI	29.26	0.57	0.11	0.87	0.11	0.68 (0.42–0.94)	0.110
	BAI	25.15	0.86	0.24	0.74	0.27	0.84 (0.66–1.00)	0.002
	WC	96.60	0.57	0.08	0.90	0.08	0.78 (0.59–0.97)	0.010
	**Women**							
	BMI	25.32	0.89	0.51	0.48	0.62	0.68 (0.55–0.80)	0.070
	BAI	34.70	0.67	0.23	0.75	0.24	0.77 (0.63–0.92)	0.005
	WC	85.65	0.89	0.43	0.56	0.52	0.74 (0.63–0.86)	0.010
**General**								
	**Men**							
	BMI	28.33	0.51	0.22	0.73	0.24	0.70 (0.63–0.77)	<0.001
	BAI	28.75	0.51	0.19	0.76	0.20	0.69 (0.60–0.77)	<0.001
	WC	94.15	0.55	0.28	0.67	0.31	0.71 (0.63–0.78)	<0.001
	**Women**							
	BMI	30.18	0.62	0.18	0.76	0.20	0.75 (0.68–0.81)	<0.001
	BAI	36.65	0.51	0.19	0.75	0.19	0.68 (0.60–0.75)	<0.001
	WC	89.75	0.74	0.26	0.69	0.31	0.79 (0.74–0.85)*	<0.001

T2DM, type 2 diabetes mellitus; BMI, body mass index; BAI, body adiposity index; WC, waist circumference; AUC, area under the ROC curve; CI, confidence interval; PPV, positive predictive value; NPV, negative predictive value.

*WC vs BAI, *p* = 0.02.

The logistic regression analysis standardized to *z* scores showed that *z*BAI (men, OR = 6.32 [95%CI, 1.41–28.28]); (women, OR = 2.52 [95%CI, 1.08–5.87]) was a better risk predictor to T2DM than *z*WC (men, OR = 2.97 [95%CI, 1.16–7.60]); (women, OR = 1.84 [95%CI, 0.96–3.54]) and *z*BMI (men, OR = 2.70 [95%CI, 0.94–7.76]); (women, OR = 1.62 [95%CI, 0.91–2.89]) in both genders (Table 3).

**Table 3:** Risk assessment for T2DM according to zBMI, zBAI and zWC in Amerindian and General population.

AMERINDIANS	OR (95%CI), *p*value			
	Men		Women	
**zBMI**	2.70 (0.94–7.76), 0.06		1.62 (0.91–2.89), 0.10	
**zBAI**	6.32 (1.41–28.28), 0.02		2.52 (1.08–5.87), 0.03	
**zWC**	2.97 (1.16–7.60), 0.02		1.84 (0.96–3.54), 0.07	
**GENERAL POPULATION**				
**zBMI**	2.27 (1.57–3.28), <0.001		2.02 (1.62–2.53), <0.001	
**zBAI**	2.54 (1.49–4.33), <0.001		1.77 (1.37–2.29), <0.001	
**zWC**	2.03 (1.44–2.86), <0.001		2.37 (1.81–3.09), <0.001	

T2DM, type 2 diabetes mellitus; zBMI, body mass index z-score; zBAI, body adiposity index z-score; zWC, waist circumference z-score; OR, odds ratio; CI, confidence interval.

### General Population

Demographic data related to age, BMI, BAI, WC, fasting glucose, and T2DM prevalence stratified for sex are summarized in Table 1.

Cutoffs, sensitivity, specificity, PPV, NPV, and AUC are reported in Table 2. In men, the discriminatory powers of anthropometric indices in the risk assessment for T2DM were not different. However, in women, the AUC of WC was significantly higher than that of BAI (0.79 vs 0.68, *p* = 0.02, respectively). In addition, different from the scenario observed in Amerindians, both the sensitivity and specificity of the different indices were very similar across indices and sexes, with the exception of a higher sensitivity observed for WC in women.

The logistic regression analysis standardized to *z* scores showed that in men, *z*BAI (OR = 2.54 [95%CI, 1.49–4.33]) was a better risk predictor to T2DM than *z*BMI (OR = 2.27 [95%CI,1.57–3.28]) and *z*WC (OR = 2.03 [95%CI, 1.44–2.86]). However, in women, *z*WC (OR = 2.37 [95%CI, 1.81–3.09]) was a better risk predictor to T2DM than *z*BMI (OR = 2.02 [95%CI, 1.62–2.53]) and *z*BAI (OR = 1.77 [95%CI, 1.37–2.29]) (Table 3).

## Discussion

Several studies have focused on the relationship between anthropometric indices of adiposity and DM risk. Wei et al [14], studying 721 Mexican-Americans aged 25–64 years, showed that WC was a better risk predictor for T2DM than BMI, independently of age and sex. Corroborating such results, Stevens et al [15], studying 12,814 African Americans and white participants aged 45–64 years, showed that AUC for WC was higher than for BMI in African men and women. On the other hand, Tulloch-Reid et al [16], studying Pima Indians, showed that the AUC was significantly larger for BMI than for either WC or WHR. Finally, Vazquez et al [5] through a meta-analysis involving 32 studies, showed that the pooled relative risks for the incidence of DM was similar for WC, BMI, and WHR.

Regarding the discriminatory power of anthropometric indices in the risk assessment for T2DM, our results do not corroborate the findings of Wei et al [14] and Stevens et al [15]: the AUC of WC was similar to BMI in both populations (general and Amerindian). However, in the general population, the AUC of WC was higher than that of BAI in women. The findings of Tulloch-Reid et al [16] showed that BMI, a representative index of general obesity, has a higher discriminatory power in the prediction of T2DM than WC, a representative index of the abdominal obesity, in Pima Indians. Differently, in the Amerindian population of our study, the BAI, a representative index of general body fat, has a lower discriminatory power than WC in women. Thus, it becomes clear that ethnic differences may influence the discriminatory power of several anthropometric indices in the risk assessment for DM.

After the study by Bergman et al [7], several investigations have shown the role of BAI, compared to other anthropometric indices of adiposity (BMI, WC, and WHR), in body fat assessment [4], [17] and an association with cardiovascular risk factors [18], [19]. However, studies related to risk assessment for DM are still insufficient. Recently, Schulze et al [20], studying approximately 36,368 individuals of both sexes taking part in the KORA and EPIC-postdam studies, showed that BAI was associated more with DM risk compared with BMI, while WC was shown to be the strongest predictor. Corroborating, partially, such results, Talaei et al [21], studying 2981 individuals of the Iranian population for a period of seven years, showed that waist-to-height ratio (WHtR) and BMI were better than BAI in the prediction of T2DM. On the other hand, our results show that *z*BAI is superior to *z*BMI and *z*WC in the risk assessment for T2DM in the Amerindian population and in men in the general population. Nevertheless, in women in the general population, both AUC and OR for zWC were superior to BAI in discriminatory power and risk assessment of T2DM, respectively. These data could, partially, be explained by a higher average age of women in the general population compared with women in the Amerindian population, which suggests less influence of ovarian hormones on cardiometabolic disorders associated with visceral fat [22].

Part of the controversial results may be explained by the use of different statistical methods. In our study, the risk assessment for T2DM was measured by logistic regression analysis regarding continuous variables (BMI, BAI, and WC) standardized to *z* score, while Schulze et al [20] estimated the risk for DM by comparing quintiles of the anthropometric features. In addition, factors such as ethnicity and distinct DM prevalence among the populations may affect the predictive power of the anthropometric indices of adiposity.

Our study has some limitations. First, we did not perform a BAI internal validation aiming to establish its capacity to assess the body fat in the study populations. For such, it would be necessary to compare BAI with some other method capable to accurately measuring the body fat percentage, as well as dual-energy X-ray absorptiometry (DEXA). Second, it would be interesting to associate these anthropometric indices (WC, BMI, and BAI) to methods able to assess the degree of insulin resistance, as well as the HOMA-IR.

In summary, BAI may be suggested as a better risk predictor of T2DM than both BMI and WC in the Amerindian population and in men belonging to the general population. However, in women belonging to the general population, WC was superior to BMI and BAI in the prediction of T2DM. Thus, it is plausible to surmise that BAI is a useful tool for the T2DM risk assessment in admixture populations.
